# A chimeric yellow fever-Zika virus vaccine candidate fully protects against yellow fever virus infection in mice

**DOI:** 10.1080/22221751.2020.1730709

**Published:** 2020-03-02

**Authors:** Dieudonné Buh Kum, Robbert Boudewijns, Ji Ma, Niraj Mishra, Dominique Schols, Johan Neyts, Kai Dallmeier

**Affiliations:** KU Leuven Department of Microbiology, Immunology and Transplantation, Rega Institute, Laboratory of Virology and Chemotherapy, Leuven, Belgium

**Keywords:** YFV-17D, chimeric flavivirus vaccine, CD8^+^ T cells, live-attenuated vaccines, non-neutralizing antibodies

## Abstract

The recent Zika virus (ZIKV) epidemic in the Americas, followed by the yellow fever virus (YFV) outbreaks in Angola and Brazil highlight the urgent need for safe and efficient vaccines against the ZIKV as well as much greater production capacity for the YFV-17D vaccine. Given that the ZIKV and the YFV are largely prevalent in the same geographical areas, vaccines that would provide dual protection against both pathogens may obviously offer a significant benefit. We have recently engineered a chimeric vaccine candidate (YF-ZIKprM/E) by swapping the sequences encoding the YFV-17D surface glycoproteins prM/E by the corresponding sequences of the ZIKV. A single vaccine dose of YF-ZIKprM/E conferred complete protection against a lethal challenge with wild-type ZIKV strains. Surprisingly, this vaccine candidate also efficiently protected against lethal YFV challenge in various mouse models. We demonstrate that CD8^+^ but not CD4^+^ T cells, nor ZIKV neutralizing antibodies are required to confer protection against YFV. The chimeric YF-ZIKprM/E vaccine may thus be considered as a dual vaccine candidate efficiently protecting mice against both the ZIKV and the YFV, and this following a single dose immunization. Our finding may be particularly important in the rational design of vaccination strategies against flaviviruses, in particular in areas where YFV and ZIKV co-circulate.

## Introduction

The yellow fever virus (YFV) belongs, together with other medically important viruses such as the dengue (DENV), the Japanese encephalitis (JEV), the West Nile (WNV), the Zika (ZIKV) and the tick-borne encephalitis virus (TBEV), to the genus Flavivirus (family *Flaviviridae*). These viruses are transmitted by arthropod vectors, in the case of the YFV, DENV and ZIKV prominently by *Aedes* mosquitoes affecting the lives of hundreds of millions of people around the globe. Vaccination has proven the most efficient and cost-effective intervention strategy to prevent flavivirus infections, especially in areas where high mosquito densities impair vector control. However, licensed vaccines available only for the YFV, JEV and TBEV. Recently, after an explosive outbreak of ZIKV in the Americas [1], a YFV outbreak was reported to encroach on cities in Angola and later spread to the Democratic Republic of Congo and Uganda [2,3]. During the epidemic, more than 2000 YFV cases including >500 deaths were confirmed as well as >4000 epizootic cases involving non-human primates [3]. Owing to this unprecedented outbreak, a high demand for YF vaccination in vulnerable populations forced the WHO to deplete its reserve stockpile of YF vaccines twice and to implement a fractional dosing (1/5) strategy for the vaccine to meet the huge demand [2,3].

About 47 countries across the Americas and Africa are located in YFV risk zones [4]. Despite the availability of an efficient and safe vaccine (YFV-17D) that was developed in the 1930s [5], the disease remains a huge public health burden. YFV-17D is arguably one of the most successful vaccines ever made [6,7] with over 850 million doses being administered since its introduction. This vaccine elicits robust immune responses as early as 10 days post vaccination and may confer likely life-long protective immunity in vaccinees [8,9]. Neutralizing antibodies have been shown to be sufficient to confer protection against lethal YFV challenge in non-human primates [10,11]. Whether or not CD8^+^ T cells play a role in the protective efficacy of the vaccine remains elusive and debatable [6,11]. On the contrary, a number of studies in mice have shown the primordial role of CD4^+^ T cells during initial YFV infection [6,11,12].

To address the functional role of YFV-17D antigenic determinants in conferring protection against a lethal challenge, we made use of our recently engineered chimeric ZIKV vaccine candidate that consists of the YFV-17D backbone from which the structural genes (prM/E) have been replaced by the corresponding sequence of a ZIKV isolate (YF-ZIKprM/E) [13]. Despite being strongly attenuated in mice compared to the parental YFV-17D, this vaccine virus proved very efficient against stringent ZIKV challenge in various mouse models. In addition, the pups of vaccinated female mice were even completely protected against direct intraplacental inoculation with a high titre of the ZIKV. As little as 10^2^ PFU was sufficient to elicit robust neutralizing antibodies (nAb) against the ZIKV as early as 7 days post vaccination [13]. The vaccine virus was also shown to elicit multi-functional CD4^+^ and CD8^+^ T cell responses against the ZIKV structural as well as the YFV-17D non-structural proteins [13]. This raises the question as to whether such vigorous (memory) T cell responses against the YFV-17D non-structural proteins may confer protection against a lethal YFV challenge. Generally, the structural proteins, principally the E protein, of flaviviruses are the main targets to which neutralizing antibodies are elicited that are believed to be required and sufficient for protection against a lethal flavivirus challenge [10–12]. We here demonstrate that a YF-ZIKprM/E which thus lacks the envelope glycoprotein of YFV, fully protects mice against a lethal YFV challenge without eliciting YFV-specific neutralizing antibodies. The observed dual protection against ZIKV [13] and YFV may particularly help rationalizing vaccine design against other flaviviruses especially for areas where they co-circulate.

## Methods

### Cells

Vero E6 and BHK-21J cells were maintained in Minimum Essential Medium (MEM Invitrogen) supplemented with 10% foetal bovine serum (FBS), 2 mM L-glutamine (Gibco, Belgium), 1% sodium bicarbonate (Gibco), and incubated at 37°C in the presence of 5% CO_2_.

### Viruses

YF-ZIKprM/E is a derivative of the YFV-17D vaccine expressing an Asian-lineage ZIKV prM/E envelope [13]. YFV-17D-Nluc is a YFV-17D derivative with a Nanoluciferase reporter gene cassette inserted into the YFV-17D polyprotein downstream of codon 22. The construction of YFV-17D-Nluc is described elsewhere (Schmid et al, manuscript in preparation). Working stocks of YFV-17D were generated by passaging Stamaril® (Sanofi-Pasteur MSD, Brussels, lot H5105) purchased from the pharmacy of the University Hospital Leuven once on BHK-21J cells and twice on Vero E6 in 2% FBS MEM. Supernatants were harvested when virus-induced cytopathic effect became evident, cleared by centrifugation (3000 g for 10 min) and stored at −80°C.

### Mice

AG129 mice (129Sv/Ev mice deficient in interferon-α/β and -γ receptors; B&K Universal, Marshall Bio Resources, UK) and *ifnar1*^−/−^ mice (interferon-α/β receptor deficient C57BL/6 mice) were bred at the Experimental Animal Facilities of KU Leuven, Belgium. Wild-type C57BL/6 mice were purchased from Janvier, France. All animal experiments strictly followed Belgian and FELASA (Federation of European Laboratory Animal Science Associations) guidelines, in accordance with the Ethical Committee of the Animal Research Centre of KU Leuven (project number P140-2016). Six to eight weeks old mice were either sham-vaccinated with 2% FBS MEM or vaccinated intraperitoneally (i.p.) with 1 × 10^4^ plaque-forming units (PFU) of YF-ZIKprM/E prior to i.p. infection/challenge with doses of YFV-17D as individually indicated (range 10^3^–10^4^ PFU). Euthanasia (with 150 mg/kg i.p. Dolethal) was considered when animals showed overt signs of disease such as ≥20% weight loss, paralysis, hunch posture, ruffled fur, watery/sunken eyes. As YFV-17D does not readily replicate in wild-type mice [6], 2 mg of the type-1 interferon receptor blocking antibody, MAR1-5A3 (Leinco, #I-1188) [26], was administered i.p. 1 day prior to immunization and challenge of C57BL/6 mice.

### T cell depletion and adoptive transfers

For T cell depletion studies, AG129 mice were either sham-vaccinated or vaccinated as before. At days −2 and 0 prior to i.p. challenge with YFV-17D, mice were i.p. administered 0.5 mg of either anti-mouse CD4 (Clone GK1.5, Leinco) or anti-mouse CD8a (Clone 53-6.7, Leinco) depleting antibodies or a combination of both. For adoptive transfer of serum or T cells, AG129 and *ifnar*^−/−^ mice (serving as donors) were vaccinated with either 1 × 10^4^ PFU of YFV-17D-NLuc, YF-ZIKprM/E or sham-vaccinated. AG129 mice were boosted twice (days 28 and 42 after primary vaccination) and bled twice weekly after the last boost, the respective sera were pooled and stored at −80°C. Ten weeks after the first vaccination, mice were euthanized, blood and spleens collected, pooled and prepared for transfer. Spleens were pushed through 70 µm cell strainers (Falcon) to generate single cell suspensions for injection of 1–5 × 10^7^ splenocytes via the tail vein. For serum transfer, mice were injected i.p. with 300 µl of sera on days −1, 2 and 6 pre- and post-challenge, respectively.

### RNA extraction and qRT-PCR

To determine YFV-17D RNA loads, mice were euthanized, serum and organs (in RNAlater, Thermo Fisher) were collected and stored at −80°C prior to RNA extraction essentially as previously described for ZIKV [13,27]. Quantitative RT-PCR of YFV-17D was performed using primers and probes (Table S1) as described before [28]. Quantitative RT-PCR was performed using the BioRad iTaq Universal Probe One-Step kit (#172-5141) and samples were run using a Roche LightCycler^®^ 96 instrument. RNA values were calculated based on a standard curve generated by serial dilution of a plasmid DNA standard.

### Indirect immunofluorescence assay (IIFA)

Seroconversion assays to determine total virus-binding antibodies (bAb) were performed using an in-house IIFA as earlier described [13]. Briefly, Vero E6 cells were infected with ZIKV BeH819015 [29], or YFV-17D at a multiplicity of infection of 0.1 and incubated for 2 days to allow > 95% infection of cells. Cells were trypsinized, resuspended in 2% FBS MEM and seeded in 96-well plates to serve as antigen for the detection of antibodies in sera. Test sera were diluted serially 1:20–1:20,0000 and added to the fixed cells for 1 h prior to detection using a FITC-labelled secondary anti-mouse antibody. All downstream steps were performed as previously described [13].

### Plaque reduction neutralization assay (PRNT)

PRNT for YFV and ZKV nAb were performed as described [13]. In brief, ZIKV MR766 or YFV-17D (40–80 PFU) was pre-incubated at 37°C for 1 h with or without a dilution series of sera before the virus-antibody complexes were added to monolayers of BHK-21J cells in 24-well plates. After 1 h at 37°C, cells were washed overlaid with 0.5% low melting agarose (Invitrogen) prepared in MEM 2% FBS. After incubation at 37°C for 7 days, cells were fixed and plaques visualized by staining with crystal violet. Serum dilution that resulted in 50% reduction of plaque counts compared to untreated YFV-17D infected controls is reported as PRNT_50_.

### Measurement of pro-inflammatory cytokines and chemokines

Pro-inflammatory cytokines, namely; TNF-α, IFN-γ, IL-6, IL-18, CCL2 (MCP-1), CCL3 (MIP-1α), CCL4 (MIP-1β), CCL5 (RANTES), CCL7 (MCP-3), CCL11 (Eotaxin), CXCL1 (GRO-α), CXCL2 (MIP-2), CXCL10 (IP-10), GM-CSF, IL-1β, IL12p70, IL-13, IL-2, IL-4, and IL-5 were quantified using the ProcartaPlex Mouse Th1/Th2 & Chemokine Panel I kit [ThermoFisher, #EPX200-26090-901], performed with 20 μL of serum on a Luminex 100 instrument (Luminex).

### Antigens for T cell assays

For T cell assays following recall antigens were used; ZIKV BeH819015 or YFV-17D infected Vero E6 cell lysates, or with a ZIKV E peptide pool (JPT Peptide Technologies), or an MHC I haplotype class-restricted YFV-17D NS3 peptide [17] (sequence ATLTYRML, Eurogentec). To produce virus cell lysates, Vero E6 cells were infected with YFV-17D or ZIKV followed by serial freeze-thaw cycles two days post infection. After UV inactivation of residual viruses, lysates were used as such for the stimulation of splenocytes. Non-infected Vero E6 cell lysate was used as a negative control.

### ELISPOT

ELISPOT was performed using a mouse IFN-γ ELISPOT (ImmunoSpot MIFNG-1M/5, CTL, Germany) as described [13]. In brief, sham-vaccinated and vaccinated mouse splenocytes (4 × 10^5^ per well) were each incubated with 1 µM/peptide of the ZIKV E peptide pool, or 5 µM of the YFV-17D NS3 ATLTYRML peptide, or 50 µg/ml of the Vero E6 cell lysates at 37°C for 24 h and IFN-γ spots were developed and counted (ImmunoSpot S6 Universal Reader, CTL). Data were normalized by subtracting the number of spots from samples incubated with non-infected Vero E6 cell lysates.

### Statistical analysis

Data were analysed using GraphPadPrism v7 and presented as mean values ± standard error of mean (SEM). Mann–Whitney two-tailed test was performed for comparison between 2 groups, and one-way ANOVA with Bonferroni correction for multiple comparisons between groups. Survival between groups was compared using the Log-rank (Mantel–Cox) test. *P*-values < 0.05 indicate a statistical difference between groups: *P-values < 0.05, **P-values < 0.01, ***P-values < 0.001, ****P-values < 0.0001.

## Results

### Mice vaccinated with YF-ZIKprM/E are fully protected against YFV-17D challenge in different mouse models

**Figure 1.**
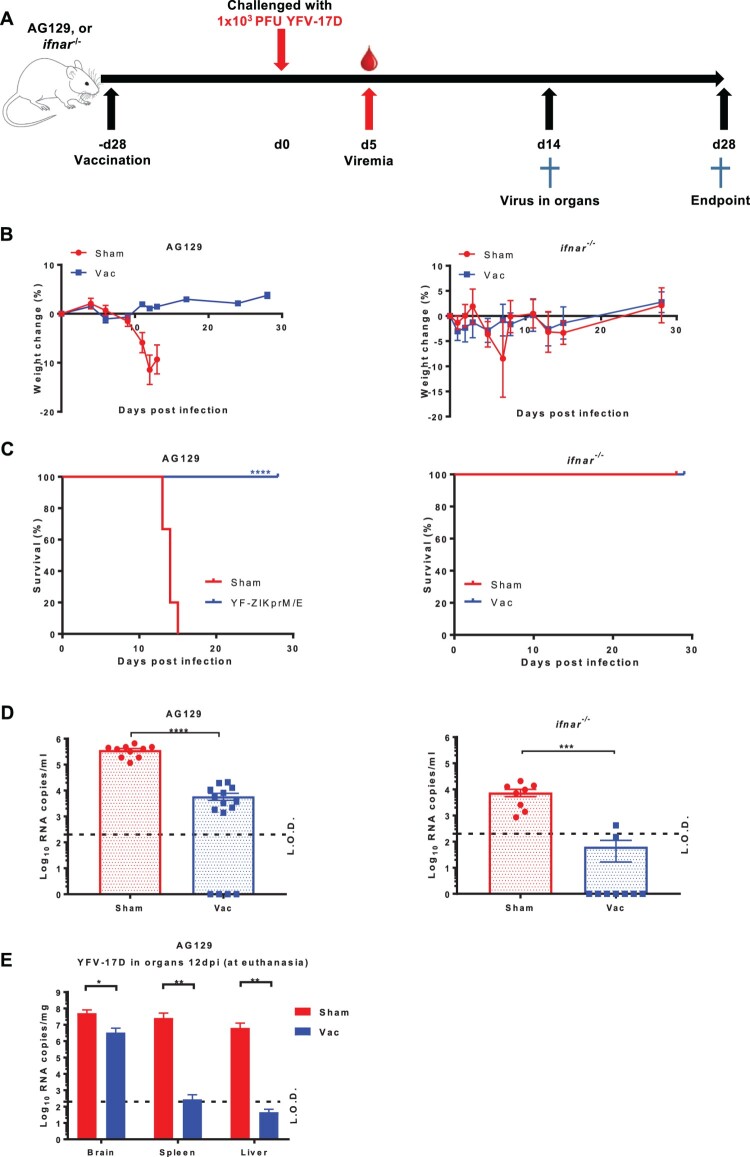
Protective efficacy of YF-ZIKprM/E against YFV-17D challenge in AG129 and *ifnar*^−/−^ mice. (A) Schematic representation of the vaccine-challenge protocol in different mouse models. AG129 and *ifnar*^−/−^ (6–8 weeks old) were randomly assigned to cages in groups of 5 and either i.p. vaccinated with 1 × 10^4^ PFU of YF-ZIKprM/E (*n* = 9 or 15) or sham-vaccinated (*n* = 10). Twenty-eight days after vaccination, mice were challenged with 1 × 10^3^ PFU of YFV-17D. Weight change (B) and survival (C) of sham-vaccinated (red circles) and YF-ZIKprM/E vaccinated (blue squares) mice following i.p. YFV-17D challenge. Challenge virus viremia was quantified by qRT-PCR at day 5 after YFV-17D challenge of AG129 and *ifnar*^−/−^ mice (D). Red circles represent sham-vaccinated while blue squares represent vaccinated mice. A fraction of asymptomatic vaccinated (*n* = 5) and symptomatic sham-vaccinated (*n* = 10) AG129 mice were euthanized and virus titres were quantified by qRT-PCR in the brain, kidney and liver (E). Data are presented as mean values ± SEM from at least 2 independent experiments (*n* = 5–10). Log-rank (Mantel-Cox) test was used to assess statistical differences in survival rates between sham-vaccinated and vaccinated mice. Mann-Whitney two-tailed test to compare viremia between sham-vaccinated and vaccinated mice. *P*-values < 0.05 were considered statistically significant. *P < 0.05, **P < 0.01, ***P < 0.001, ****P < 0.0001. Dotted lines denote the limit of detection (L.O.D.) of the assay.

Mice (either AG129 and *ifnar*^−/−^) were either vaccinated with 1 × 10^4^ PFU of the Zika vaccine candidate YF-ZIKprM/E [13] or sham-vaccinated. Twenty-eight days later mice were challenged with 1 × 10^3^ PFU of YFV-17D (Figure 1(A)). The YFV-17D strain, though attenuated for humans/primates, is known to cause virus-induced disease in *ifnar*^−/−^ mice and uniformly lethal infections in AG129 mice, and is hence established as a BSL2 surrogate for wild-type YFV infection in these models [6,13–16,30]. As expected, all sham-vaccinated mice progressively lost weight, although *ifnar*^−/−^ later recovered (Figure 1(B), S1). In line, all sham-vaccinated AG129 mice consistently developed disease and had to be euthanized 13–18 post challenge. By contrast, all YF-ZIKprM/E vaccinated AG129 mice survived the YFV-challenge (Figure 1(C)). Both YF-ZIKprM/E vaccinated and sham-vaccinated AG129 mice developed YFV viremia (as measured by qRT-PCR at day 5 post challenge) but this was significantly (>1 log_10,_ *P *< 0.0001) reduced in the vaccinated mice (sometimes to undetectable levels) as compared to the control mice (Figure 1(D)). All (9/9) of sham-vaccinated *ifnar*^−/−^ mice developed viremia whereas only 2/9 (22%) of the YF-ZIKprM/E vaccinated mice did (Figure 1(D)). At euthanasia, viral YFV-17D RNA was quantified in organs (brain, spleen, liver) of AG129 mice. For every two euthanized sham-vaccinated mice, one asymptomatic vaccinated mouse was euthanized at the same time point. Vaccinated AG129 mice had a reduction in viral load of 1 log_10_ (*P *< 0.05)_,_ 5 log_10_ and 5 log_10_ (both *P *< 0.01) in brain, spleen and liver respectively, compared to sham-vaccinated mice (Figure 1(E)). As expected, vaccination with YFV-17D-NLuc (an attenuated variant of YFV-17D that does not cause disease in AG129 mice anymore) protected AG129 mice against disease and mortality from a subsequent YFV-challenge (Figure S2).

**Figure 2.**
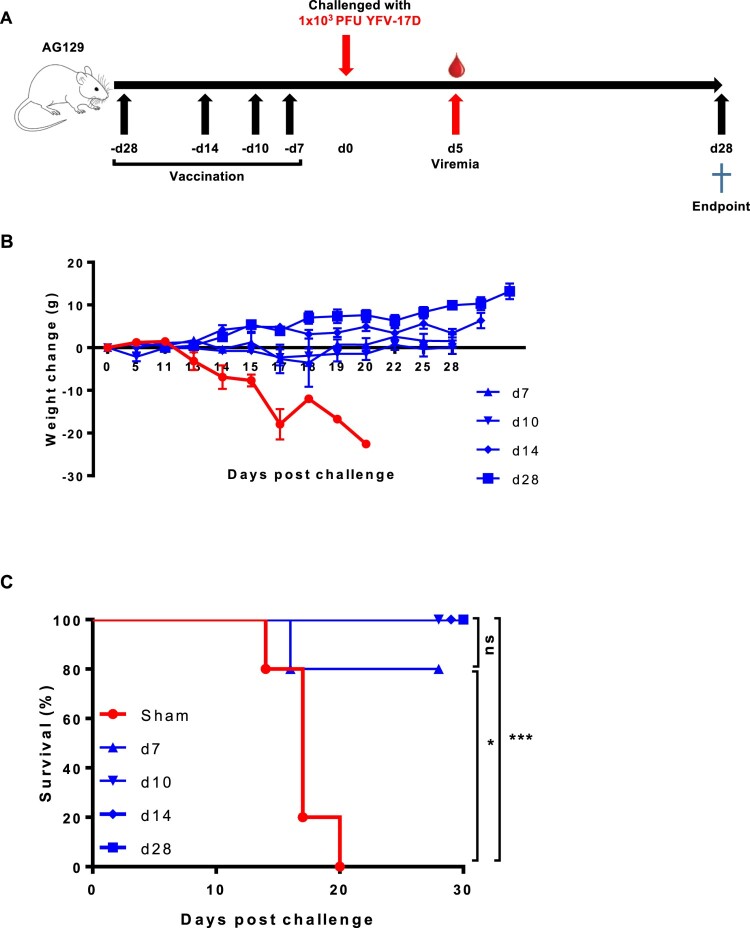
Time to protection from YFV-17D-induced weight loss and mortality (i.e. euthanasia) after YF-ZIKprM/E vaccination in AG129 mice. (A) Schematic representation of the time to protection of YF-ZIKprM/E against YFV-17D in AG129 mice. (B) Groups of 6–8 weeks old AG129 mice were either sham-vaccinated (*n *= 5) or i.p. vaccinated (*n *= 5/group) with 1 × 10^4^ PFU of YF-ZIKprM/E at different time points (days 28, 14, 10, 7) prior to challenge with 1 × 10^3^ PFU of YFV-17D. Weight and the general condition was monitored for the next 28 days. (C) Survival of sham-vaccinated (red) and YF-ZIKprM/E vaccinated (blue) mice following i.p. challenge with YFV-17D. Data presented as means ± SEM. Log-rank (Mantel-Cox) test was used to assess statistical differences in survival rates between sham-vaccinated and YF-ZIKprM/E vaccinated mice. *P*-values < 0.05 were considered statistically significant. *P < 0.05, ***P < 0.001, ns = non-significant.

To explore how soon after YF-ZIKprM/E vaccination AG129 mice were protected against YFV-17D challenge, mice (*n* = 5 each) were either sham-vaccinated or vaccinated with 1 × 10^4^ PFU of YF-ZIKprM/E at either 28, 14, 10, or 7 days prior to challenge with 1 × 10^3^ PFU YFV-17D (Figure 2(A)). All control mice developed disease (Figure 2(B)) and had to be euthanized [mean days to euthanasia (MDE) of 17 ± 3] (Figure 2(C)). All vaccinated mice were completely protected from YFV-17D-induced weight loss (Figure 2(B)) and mortality (Figure 2(C)) except 1 mouse which was vaccinated only 7 days prior to challenge.

### YF-ZIKprM/E does not elicit neutralizing antibodies against YFV-17D

**Figure 3.**
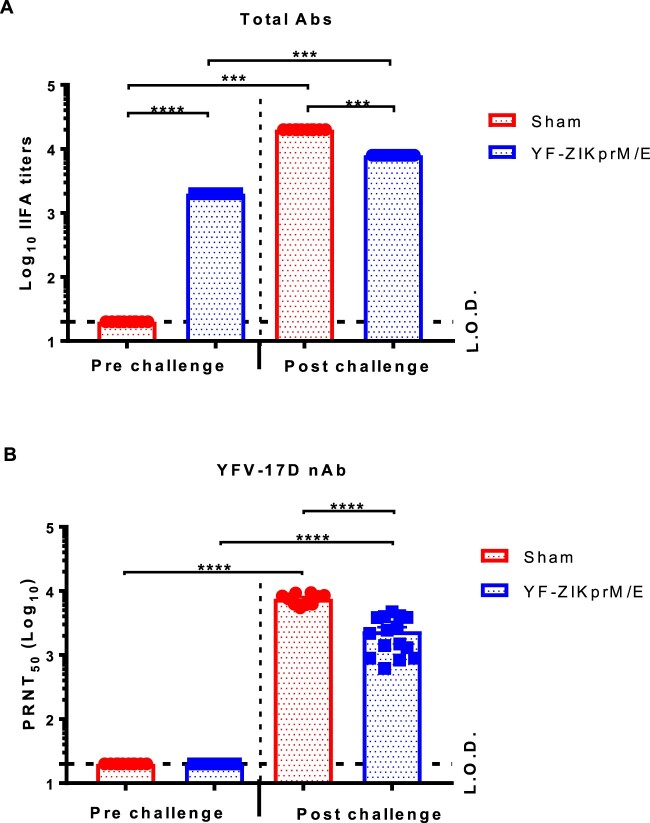
Serum from YF-ZIKprM/E vaccinated mice does not neutralize YFV-17D *in vitro*. Serum samples were collected from sham-vaccinated and YF-ZIKprM/E vaccinated AG129 mice pre- and 14 days post- i.p. challenge with YFV-17D. (A) Total binding antibody (bAb) titres of sham-vaccinated (red circles, *n* = 10) and YF-ZIKprM/E vaccinated (blue squares, *n* = 15) sera pre- and post- YFV-17D challenge. (B) Neutralization potential of sham-vaccinated (red circles, *n* = 10) and YF-ZIKprM/E vaccinated (blue squares, *n* = 15) sera pre- and post- YFV-17D challenge. Data are presented as mean values ± SEM of at least 2 independent experiments (*n* = 5–10). Mann-Whitney two-tailed test was performed to assess statistical differences between groups. *P*-values < 0.05 were considered statistically significant. ***P < 0.001, ****P < 0.0001. Dotted line denotes the limit of detection (L.O.D.) of the assay.

To study whether serum from YF-ZIKprM/E vaccinated mice can neutralize YFV-17D, AG129 mice were vaccinated with 1 × 10^4^ PFU of YF-ZIKprM/E. Sera were collected pre- and post-vaccination, and the titres of total binding (bAb) and of nAb against YFV-17D was determined by IIFA and PRNT, respectively. All vaccinated mice seroconverted to high titres of YFV-17D-specific bAbs (Figure 3(A)), but no nAbs against this virus were detected (Figure 3(B)). After YFV-17D challenge, both sham-vaccinated and vaccinated animals had (at day 12 post challenge) high levels of nAbs against YFV-17D, with higher levels in the sham-vaccinated group (*P *< 0.0001). Thus YF-ZIKprM/E elicits high titres of antibodies against YFV-17D that, however, are likely not cross-reactive with the YFV envelope and are hence able to neutralize YFV infection.

**Figure 4.**
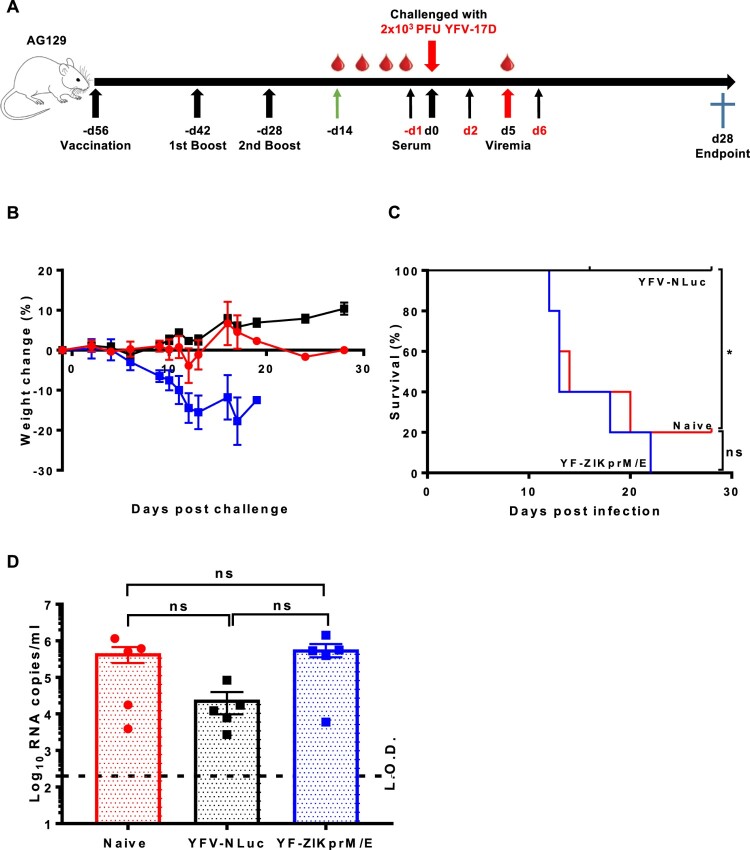
Adoptive serum transfer from YF-ZIKprM/E vaccinated mice fails to protect against YFV-17D challenge in AG129 mice. (A) Schematic presentation of adoptive serum transfer in AG129 mice. Mice were either sham-vaccinated (*n* = 5) or vaccinated with either 1 × 10^4^ PFU of Nanoluciferase expressing YFV-17D (YFV-17D-NLuc) (*n* = 5) or YF-ZIKprM/E (*n* = 5) boosted twice with the same doses. Respective sera were collected and pooled and 300 µl of each serum pool injected three times (day −1, 2 and 6) i.p. into 6-weeks old AG129 mice (*n* = 5 per group) prior to i.p. challenge with 2 × 10^3^ PFU of YFV-17D and a 28 days follow-up. Weight change (B) and survival (C) of AG129 mice that received sera from sham-vaccinated (naive, red circles), YFV-17D-NLuc-vaccinated (black squares) or YF-ZIKprM/E-vaccinated (blue squares). (D) Viremia was quantified by qRT-PCR (5 days post challenge) in mice that received sera from naive (red circles, *n* = 5), YFV-17D-NLuc (black squares, *n* = 5) and YF-ZIKprM/E (blue squares, *n* = 5). Each data point represents a single mouse. Log-rank (Mantel-Cox) test was used to measure statistical differences in survival rates between different groups. Data present mean values with error bars indicating SEM. To compare viremia between groups, two-way ANOVA with Bonferroni correction was used and *P*-values < 0.05 were considered statistically significant. *P < 0.05. ns = not significant. Dotted line denotes the limit of detection (L.O.D.) of the assay.

To further corroborate this finding, it explored whether adoptive transfer of sera from YF-ZIKprM/E vaccinated mice had an impact on YFV-17D challenge in AG129 mice. To that end, 300 µl of pooled sera from AG129 mice that had been vaccinated with YF-ZIKprM/E or sham or from mice that had been vaccinated with YFV-17D-NLuc was transferred to naive AG129 mice by i.p. injection. Serum transfer was performed either 1 day before and days 2 plus 6 after i.p. challenge with 2 × 10^3^ PFU of YFV-17D (Figure 4(A)). As expected, transfer of serum from YFV-17D-NLuc vaccinated mice fully protected (100%) against challenge virus-induced disease and mortality (Figure 4(B, C)) and lowered viremia by about 1 log_10_ (consistently, though not statistical significant by ANOVA) (Figure 4(D)). Thus, nAb is sufficient to confer full protection against YFV-17D infection. In contrast, transfer of serum from YF-ZIKprM/E-vaccinated or from naive mice did not result in any protective effect after challenge (Figure 4(B–D)). Pooled serum from YF-ZIKprM/E-vaccinated mice contain high levels of total bAb titres (Figure S3A) with high neutralizing activity against ZIKV (Figure 2(B)) but not against YFV-17D (Figure S3C). Likewise, serum from YFV-17D-NLuc-vaccinated mice had no measurable neutralization against ZIKV (Figure S3B) but a strong neutralization against YFV-17D (Figure S3C). Thus, antibodies against ZIKV prM/E do not cross-neutralize YFV and *vice versa*, neither *in vitro* nor *in vivo* as demonstrated by PRNT and YFV-17D challenge, respectively.

### YF-ZIKprM/E elicits robust T cell responses in AG129 and *ifnar*^−/−^ mice

**Figure 5.**
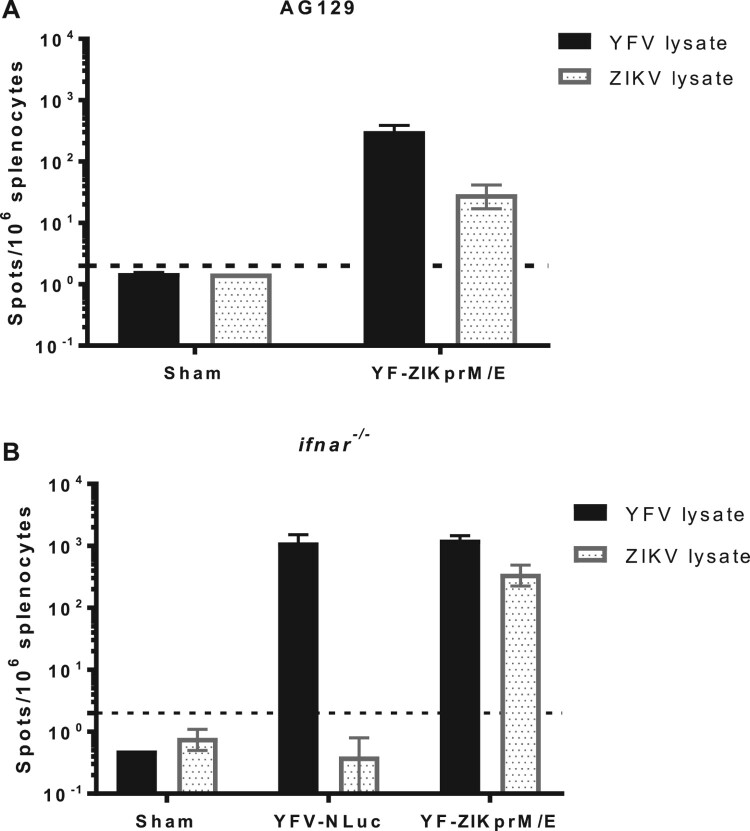
*Ex vivo* stimulation of splenocytes from YF-ZIKprM/E and sham-vaccinated mice. AG129 or *ifnar*^−/−^ mice were either sham-vaccinated or vaccinated with either 1 × 10^4^ PFU of YFV-17D-NLuc or YF-ZIKprM/E. AG129 mice were boosted twice with the same dose (used for vaccination) in intervals of two weeks and later euthanized 10 weeks after the initial vaccination. *Ex vivo* stimulation of AG129 (A) and *ifnar*^−/−^ (B) mouse splenocytes with either YFV-17D or ZIKV BeH819015 [29] total cell lysates. Data presenting mean values ± SEM of biologically independent samples (*n* = 5 per group). Dotted line denotes the limit of detection (L.O.D.) of the assay.

To dissect the functional role of YF-ZIKprM/E antigens in conferring protection against YFV, AG129 or *ifnar*^−/−^ mice were either sham-vaccinated or vaccinated with 1 × 10^4^ PFU of YF-ZIKprM/E. Ten weeks post-immunization, mice were euthanized as donors for splenocytes. Respective T cell responses were quantified for IFN-gamma ELISpot by *ex vivo* stimulation with different antigens, i.e. cell lysates of cells infected with either YFV-17D, ZIKV (Figure 5(A, B)), or peptides derived from the YFV-17D NS3 and NS4B proteins (Figure S4, Table S2). Robust T cell responses were observed in splenocytes derived from YF-ZIKprM/E-vaccinated mice (Figure 5(A, B)). The T cell responses (number of spots) were on average 5 times higher in *ifnar*^−/−^ (Figure 5(B)) than in AG129 mice (Figure 5(A)), underscoring the important role of intact IFN-gamma signalling for the induction of cellular immune responses. Strikingly, both in AG129 (Figure 5(A)) and *ifnar*^−/−^ (Figure 5(B)) mice, the responses were skewed towards the YFV-17D backbone. Overall, such vigourous T cell responses suggested an important functional role of cell mediated immunity in the protection against lethal YFV infection that could be induced by our original Zika vaccine candidate YF-ZIKprM/E.

### CD8^+^ but not CD4^+^ T cells play a crucial role in virus clearance in vaccinated AG129 mice

**Figure 6.**
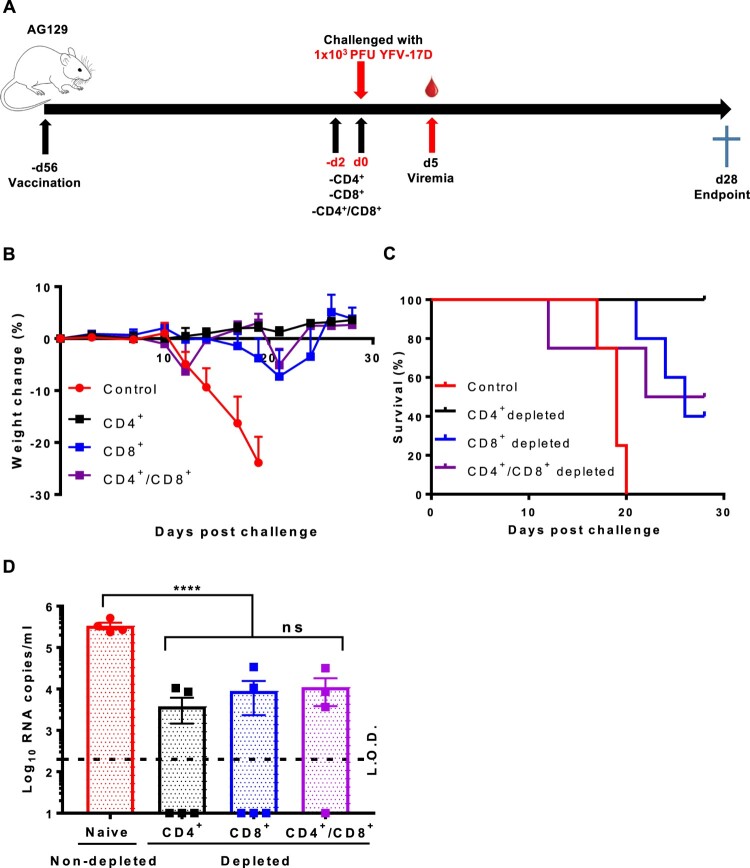
CD8^+^ T cells play a crucial role in the control and clearance of YFV-17D. (A) Schematic representation of T cell depletion in AG129 mice. Mice were either sham-vaccinated (*n* = 4) or vaccinated with 1 × 10^4^ PFU of YF-ZIKprM/E (*n* = 4–5/group). Ten weeks after vaccination, vaccinated mice were depleted of either CD4^+^ (black squares *n* = 5), CD8^+^ (blue squares, *n* = 5) or both (purple squares, *n* = 4) and challenged i.p. with 1 × 10^3^ PFU of YFV-17D. Weight change (B) and survival (C) was monitored over a period of 28 days following challenge. (D) Viremia was quantified by qRT-PCR 5 days post challenge [CD4^+^ (black squares, *n* = 5), CD8^+^ (blue squares, *n* = 5) or CD4^+^/CD8^+^ depleted (purple squares, *n* = 4)] presented as mean values ± SEM. To compare viremia between groups, two-way ANOVA with Bonferroni correction was used and *P*-values < 0.05 were considered statistically significant. ****P < 0.0001. ns = not significant. Dotted line denotes the limit of detection (L.O.D.) of the assay.

It was next studied which T cell lineages are responsible for the protection against YFV challenge in our mouse model. To that end, AG129 mice were either sham-vaccinated or vaccinated with 1 × 10^4^ PFU of YF-ZIKprM/E; ten weeks later, vaccinated mice were depleted of either their CD4^+^, CD8^+^, or both (CD4^+^/CD8^+^) cells, whereas sham-vaccinated/non-depleted mice served as controls. Three days post-challenge, blood was sampled to quantify YFV-17D viremia; mice were then further observed for 4 weeks for challenge virus-induced morbidity and mortality (Figure 6(A)). As expected, sham-vaccinated/non-depleted mice all developed disease and had to be euthanized (MDE: 18 ± 2) (Figure 6(B, C)). Depletion of CD4^+^ cells from vaccinated mice had no effect (100% survival) in AG129 mice whereas depletion of CD8^+^ reduced survival by 40% mortality (only 3/5 surviving, Figure 6(C)), suggesting that CD4^+^ cells do not play a pivotal role in protection against YFV-17D challenge. However, double depletion of CD4^+^/CD8^+^ seemed to render mice slightly more susceptible to YFV-17D infection in which disease onset and progression to disease (requiring euthanasia) appeared earlier than in CD8^+^ depleted mice (Figure 6(C)). Although the complexity of the experimental set up and the relatively small number of animals that could be used may not allow full quantitative assessment. Intriguingly, viremia was significantly reduced in vaccinated and depleted mice (regardless of the subset of T cells depleted) as compared with sham-vaccinated/non-depleted mice. All together these data suggest that, CD4^+^ cells, although not sufficient to confer protection may supplement the function of CD8^+^ cells during YFV infection.

### YF-ZIKprM/E vaccination results in significant reduction in cytokine induction following YFV-17D challenge

**Figure 7.**
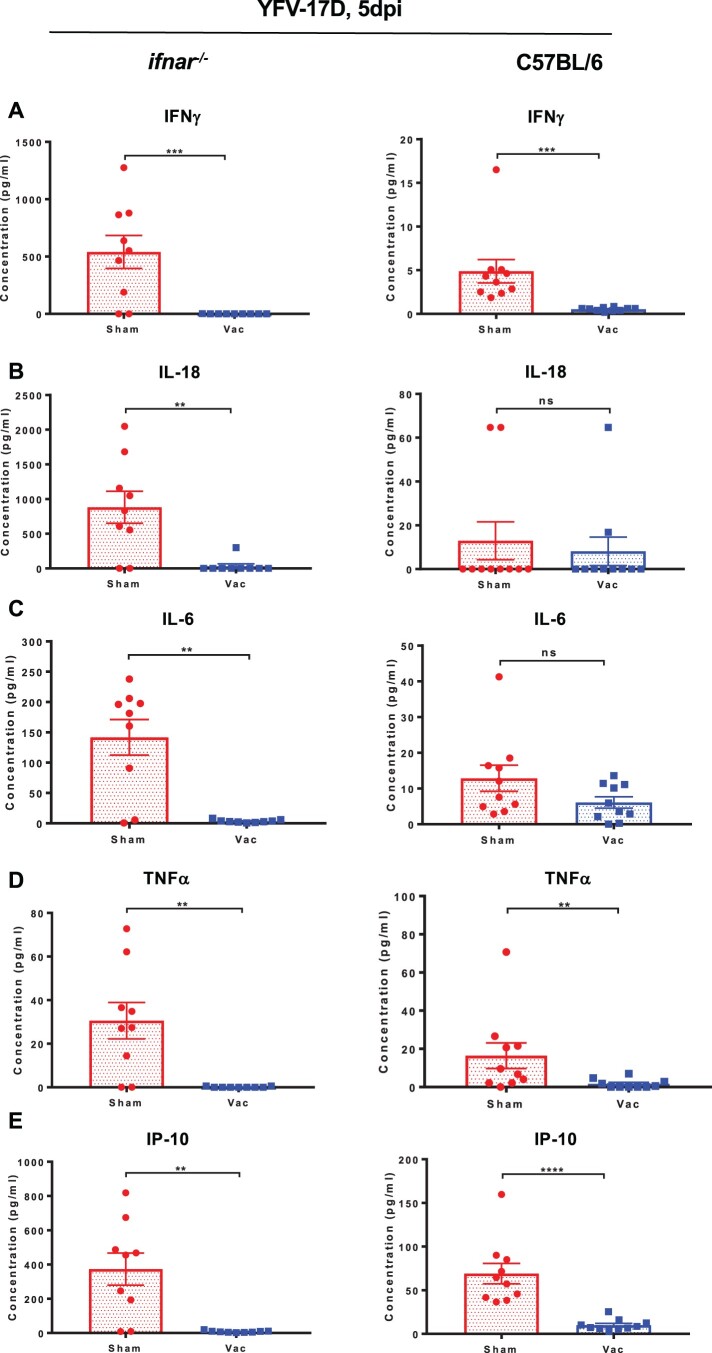
Cytokine profile of vaccinated and non-vaccinated mice challenged with YFV-17D. Wild-type C57BL/6 or *ifnar*^−/−^ mice were either sham-vaccinated with MEM 2% FBS or vaccinated with 1 × 10^4^ PFU of YF-ZIKprM/E and challenged 28 days post vaccination. C57BL/6 mice were i.p. administered 2 mg of the MAR1-5A3 1 day prior to challenge. For cytokine measurements, animals were bled by submandibular puncture 5 days post challenge. For YFV-17D challenge, sham-vaccinated (red circles, *n* = 9) or vaccinated (blue squares, *n* = 9) *ifnar*^−/−^, and sham-vaccinated (red circles, *n* = 10) or vaccinated (blue squares, *n* = 10) C57BL/6 were challenged i.p. with 1 × 10^4^ PFU of YFV-17D. Levels of interferon-gamma (A), IL-18 (B), IL-6 (C), TNF-alpha (D), IP-10 (E) and others (Figure S5) were measured in sham-vaccinated (red circles) or vaccinated (blue squares) mice 5 days post challenge. Data presented as means ± SEM. Mann-Whitney two-tailed test was performed to ascertain differences between sham-vaccinated and vaccinated mice. *P*-values < 0.05 were considered statistically significant. *P < 0.05, **P < 0.01, ***P < 0.001, ****P < 0.0001.

Elevated cytokine levels in blood have been reported to correlate with flavivirus replication in both mice and humans [13–16,27,30]. To study the extent to which YF-ZIKprM/E vaccination affects induction of cytokines after YFV challenge, *ifnar*^−/−^ and wild-type C57BL/6 mice were either sham-vaccinated or vaccinated with 1 × 10^4^ PFU of YF-ZIKprM/E. At 5 days after vaccination, they were challenged with 1 × 10^4^ PFU of YFV-17D. Five days after challenge, levels of IFN-gamma (Figure 7(A)), IL-18 (Figure 7(B)), IL-6 (Figure 7(C)), TNF-alpha (Figure 7(D)), IP-10 (Figure 7(E)) and other cytokines (Figure S5(A, B)) were measured. Cytokine levels were markedly decreased in vaccinated mice further corroborating the observed protective activity against YFV challenge virus replication.

## Discussion

The recent Zika virus (ZIKV) epidemic in the Americas, followed by the yellow fever virus (YFV) outbreaks in Angola and Brazil highlights the urgent need for safe and efficient vaccines against the Zika virus as well as a much greater capacity to produce the yellow fever virus vaccine. Given that the ZIKV and YFV are largely prevalent in the same geographical area, vaccines that would be able to protect against both pathogens at the same time might offer a significant benefit.

The role of nAbs as a surrogate of protection against YFV infections [i.e. nAb being mechanistically linked to protection [31]], is widely accepted, and nAbs often being considered sufficient and required [10,11]. However, also in the presence of nAb and CD4^+^ T cells, CD8^+^ T cells have also been demonstrated to play a substantial, yet dispensable, role and to complement antibodies in protecting against YFV [11].

We here demonstrate that a ZIKV vaccine candidate vaccine that consists of the YFV-17D backbone from which the prM/E genes have been replaced by the corresponding genes of a ZIKV isolate (Asian lineage), though highly attenuated compared to parental YFV-17D [13], confers complete protection against lethal YFV-17D challenge in an AG129 mouse model [30]. Importantly, this protection is achieved without eliciting (detectable levels of) neutralizing Abs. This was also corroborated by the observation that transfer of serum from YF-ZIKprM/E mice to AG129 did not result in protection against YFV challenge, in contrast, to transfer from YFV-17D (YFV-17D-NLuc) vaccinated mice. Such YFV-specific nAbs raised by vaccination with YFV-17D-NLuc likely protected mice more effectively from YFV-17D-induced weight loss compared to YF-ZIKprM/E vaccination (Figure S2) by directly blocking initial infection with the challenge virus.

In mice vaccinated with YF-ZIKprM/E, both the ZIKV structural and the YFV-17D non-structural proteins are targets of CD4^+^ and CD8^+^ T cell responses (Figure 5 and Figure S4). Likewise, YF-ZIKprM/E has been shown to induce multi-functional CD8^+^ T cells with a pronounced Th1 polarization (cytotoxic T lymphocytes) [13], that in turn may contribute to the protective efficacy of our Zika vaccine candidate against YFV [and possibly also ZIKV [12,13]]. The envelope (E), and non-structural proteins NS3 and NS5 of YFV proteins have been reported to contain the main immunodominant epitopes within the YFV-17D genome [17–20], and the immunogenicity of the epitopes combined within non-structural proteins to be greater than those present in the structural proteins of the YFV. One may thus assume that the combined T cell responses elicited by the non-structural proteins may, to a certain degree, confer protection against a YFV infection. Subunit vaccine candidates against ZIKV [21] and DENV [22] have been generated that target the NS1 protein of either virus. Neither vaccine candidate induces neutralizing antibodies against either ZIKV or DENV. In contrast to YF-ZIKprM/E, these NS1 targeting vaccines only conferred partial protection against challenge virus viremia [23] and mortality [22]. Likewise, vaccination using YFV NS1 has been shown to induce protective immunity in mice [32] and monkeys [33] against lethal YFV challenge, likely mediated by NS1 bAb that target YFV infected cells for Fc-mediated lysis [32]. Consequently, part of the protection induced by YF-ZIKprM/E may be due to such YFV-specific bAb that are highly prevalent in YF-ZIKprM/E vaccinated mice (Figure 3(A) and Figure S3A).

The full protection observed here with YF-ZIKprM/E, in contrast to the partial protection observed with the ZIKV and DENV NS1 vaccine candidates [21,22] may be attributed to the fact that our construct elicits a broad cell-mediated (as well as humoral) immunity against possibly all YFV non-structural proteins.

In humans, T cells are activated as early as 3 days post YFV17D vaccination [24] but CD8^+^ responses peak at day 10 [25] or 11–30 days post-immunization [24]. In the current study, we demonstrate that YF-ZIKprM/E confers protection as early as 7 days post vaccination and full protection from 10 days post vaccination onwards (Figure 2). More so, depletion of CD8^+^, but not CD4^+^ T cells in vaccinated AG129 mice rendered them susceptible to YFV-17D infection and mortality, pointing to a protective role of CD8^+^ (Figure 6(B, C)). A report on T cell transfer in B cell knock out mice lends further support to the fact that YFV-17D may induce protection mediated by CD8^+^ T cells, in the absence of any humoral response [11]. Also, depletion of CD8^+^ T cells from C57BL/6 mice vaccinated with YFV-17D resulted in increased YFV replication in the brain following intracranial challenge [11].

We also demonstrated the protective efficacy of our YF-ZIKprM/E virus vaccine candidate in *ifnar*^−/−^ as well as in immunocompetent C57BL/6 mice regarding challenge viremia (Figure 1(D), *ifnar*^−/−^) and virus-induced elevation of cytokines (Figure 7 and Figure S5). To allow the vaccine as well as the challenge virus to replicate to sufficiently high titres in C57BL/6 mice upon inoculation (since YFV does not replicate efficiently in mice), C57BL/6 mice were dosed with an interferon receptor-blocking antibody.

YFV and ZIKV co-circulate to some extent in the same geographical region. Pre-existing immunity to one flavivirus may have a beneficial (cross-protection) or adverse (enhancement) effect on the outcome of subsequent flavivirus infections. As shown here, our original Zika vaccine candidate can induce protective immunity against experimental YFV infection in several complementary mouse models. Whether or not such vaccination may be able to confer a specific protection against YFV in step-up animal models (such as non-human primates) and finally in humans remains to be investigated. In any case, such effort may require a rethinking of the surrogates of protection for yellow fever [31] beyond the established nAbs.

In conclusion, we here show that vaccination using the chimeric candidate Zika vaccine YF-ZIKprM/E can provide a remarkable protective activity against YFV in mice without eliciting measurable nAb against YFV. The observation that such a chimeric YFV-17D based Zika vaccine may possibly also confer protection against YFV is important in light of the fact that (i) both pathogens may co-circulate in the same regions and (ii) major outbreaks with the YFV (as recently happened both in Africa and Latin-America) may occur during which insufficient stockpile of the vaccine is available. More in general (iii), the same dual protective activity may apply to other, already licensed YFV-17D-based vaccines such as ChimeriVax-JE/Imojev® and CYD-TDV/Dengvaxia® that do not induce cross-reacting nAb [34] yet potent YFV-specific cellular immune responses [35,36], albeit a repurposing and off-label use in case of yellow fever vaccine shortage may not yet be warranted.

## Supplementary Material

Supplemental Material
