# Supplementary material for: A chimeric yellow fever-Zika virus vaccine candidate fully protects against yellow fever virus infection in mice
Source: Emerg Microbes Infect. 2020 Mar 2;9(1):520–33. doi: 10.1080/22221751.2020.1730709 (PMC7067203; doi:10.1080/22221751.2020.1730709)
Supplement: Supplemental Material [file TEMI_A_1730709_SM0741.docx]

**A**

**S**

**h**

**a**

**m**

**V**

**a**

**c**

**-**

**2**

**0**

**2**

**4**

**W**

**e**

**i**

**g**

**h**

**t**

**c**

**h**

**a**

**n**

**g**

**e**

**(**

**%**

**)**

**A**

**G**

**1**

**2**

**9**

**,**

**3**

**d**

**p**

**i**

**n**

**s**

**S**

**h**

**a**

**m**

**V**

**a**

**c**

**-**

**2**

**0**

**2**

**4**

**W**

**e**

**i**

**g**

**h**

**t**

**c**

**h**

**a**

**n**

**g**

**e**

**(**

**%**

**)**

**A**

**G**

**1**

**2**

**9**

**,**

**7**

**d**

**p**

**i**

**n**

**s**

***-_/-_ ifnar^-/-^*, 7dpi**

**S**

**h**

**a**

**m**

**V**

**a**

**c**

**-**

**2**

**0**

**-**

**1**

**5**

**-**

**1**

**0**

**-**

**5**

**0**

**W**

**e**

**i**

**g**

**h**

**t**

**c**

**h**

**a**

**n**

**g**

**e**

**(**

**%**

**)**

*****

**S**

**h**

**a**

**m**

**V**

**a**

**c**

**-**

**2**

**0**

**-**

**1**

**5**

**-**

**1**

**0**

**-**

**5**

**0**

**W**

**e**

**i**

**g**

**h**

**t**

**c**

**h**

**a**

**n**

**g**

**e**

**(**

**%**

**)**

***i***

***f***

***n***

***a***

***r***

**,**

**3**

**d**

**p**

**i**

*

*

**B**

# FIG S1

**FIG. S1. Weight change following YFV-17D challenge in various mouse models**. Weight change in (**A**) AG129 and (**B**) *ifnar*^-/-^ mice on days 3 and 7 post challenge with YFV-17D of sham-vaccinated AG129 (red, n = 10) and *ifnar*^-/^(red, n = 5) or YF-ZIKprM/E vaccinated AG129 (blue, n = 15) and *ifnar*^-/-^ (blue, n = 9). Data are presented as mean values with error bars indicating SEM of biologically independent samples (n = 5 – 10). Mann-Whitney two-tailed test was performed to quantify differences between sham-vaccinated and vaccinated mice. *P*-values <0.05 were considered statistically significant. **P* < 0.05, ***P* < 0.01.

**d0**

**d28**

**-**

**d28**

**Vaccination**

**(**

**Sham**

**/**

**YF**

**-**

**ZIKprM**

**/E 1x10**

**^4^**

**PFU)**

**d5**

**Viremia**

**Challenged with**

**1**

**x**

**10**

**^3^**

**PFU YFV**

**-**

**D**

**17**

**Endpoint**

**d14**

**Virus in**

**organs**


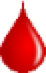

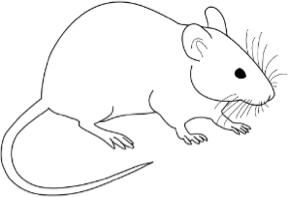


**AG129**

**A**

**B**

**Challenged with**


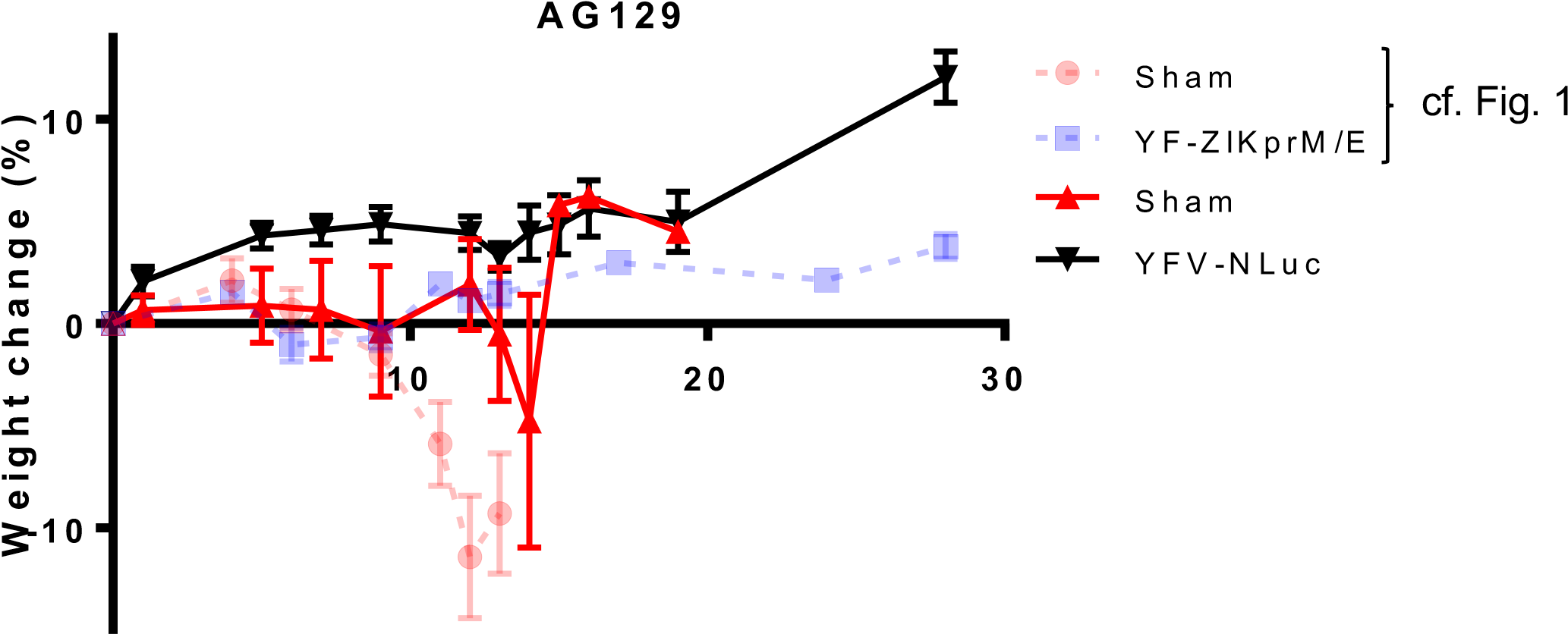

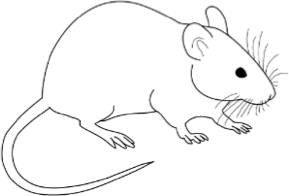
**C**

**d0**

**d28**

**10**

**x**

**1**

**3**

**PFU YFV**

**-**

**D**

**17**

**Endpoint**

**AG129**

**-**

**d14**

**Vaccination**

**(**

**Sham**

**/YFV**

**-**

**NLuc**

**,**

**5**

**x**

**10**

**4**

**PFU)**

**D ays post infection**

**D**

**A G 129**

## **** ***

**0**

**1**

**0**

**2**

**0**

**3**

**0**

**0**

**2**

**0**

**4**

**0**

**6**

**0**

**8**

**0**

**1**

**0**

**0**

**S**

**u**

**r**

**v**

**i**

**v**

**a**

**l**

**(**

**%**

**)**

**D**

**a**

**y**

**s**

**p**

**o**

**s**

**t**

**i**

**n**

**f**

**e**

**c**

**t**

**i**

**o**

**n**

**FIG S2**

S ham

cf. Fig. 1

Y F -Z IK prM /E

S ham

Y F V -N Lu c

# FIG. S2. Protective efficacy of YFV-NLuc against YFV-17D challenge in

**AG129 mice**. Schematic representation of the vaccine-challenge protocol of (**A**) YF-ZIKprM/E (cf. Fig. 1) or (**B**) YFV-NLuc vaccination. AG129 (6 – 8 weeks old) were either i.p. vaccinated with 5 x 10^4^ PFU of YFV-NLuc (n = 7) or shamvaccinated (n = 5). Fourteen days after vaccination, mice were challenged with 1 x 10^3^ PFU of YFV-17D. Weight change (**B**) and survival (**C**) of shamvaccinated (red triangles) and YFV-NLuc vaccinated (black triangles) mice following i.p. YFV-17D challenge. Data from an independent experiment with YF-ZIKprM/E (cf. Fig. 1) are represented in transparent colors. Data are presented as mean values ± SEM. Log-rank (Mantel-Cox) test was used to assess statistical differences in survival rates between sham-vaccinated and vaccinated mice. *P*-values < 0.05 were considered statistically significant. ****P* < 0.001, *****P* < 0.0001.

**A**


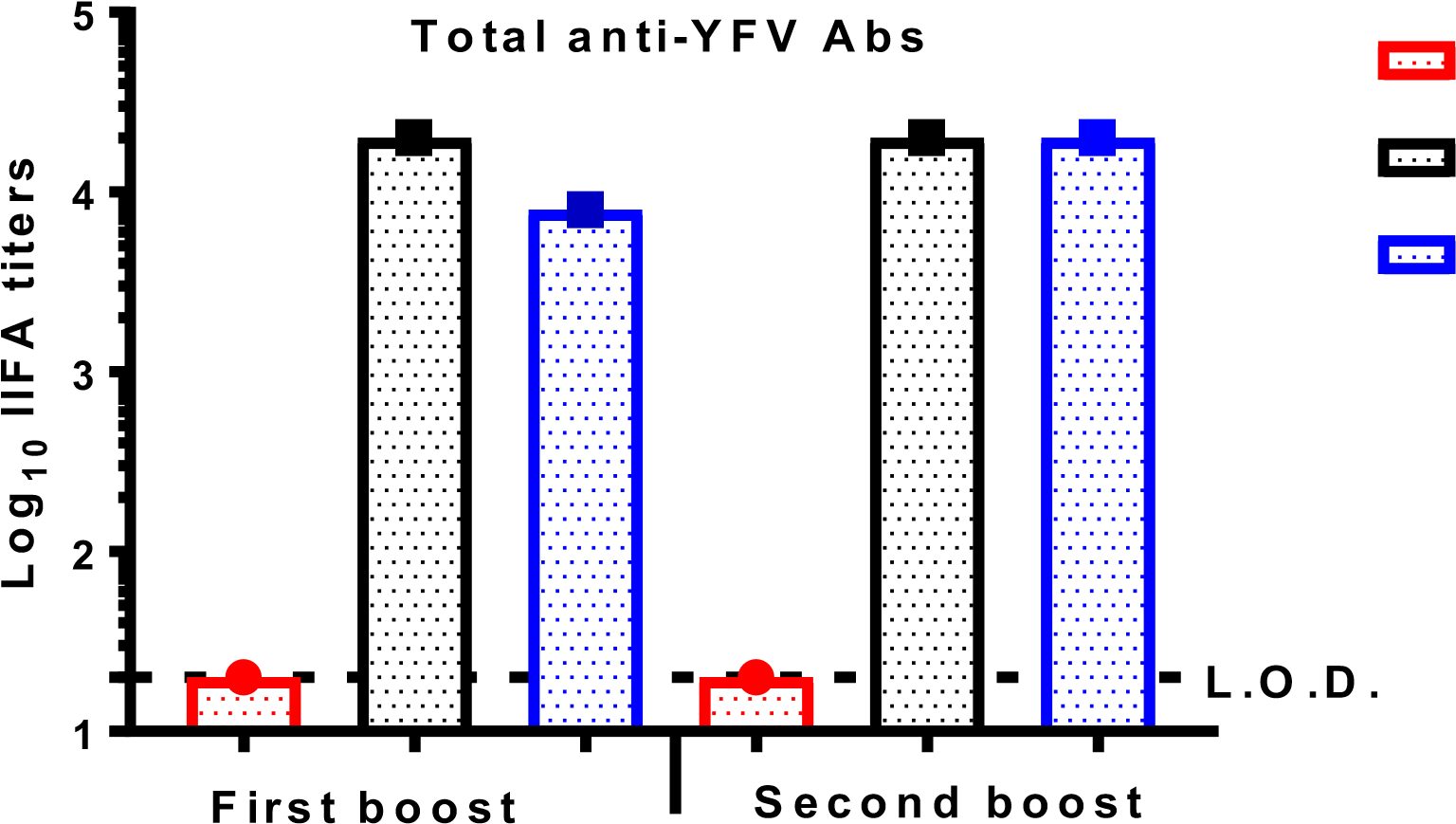
Sham

Y FV -N Luc

Y F -ZIK prM /E

**B**


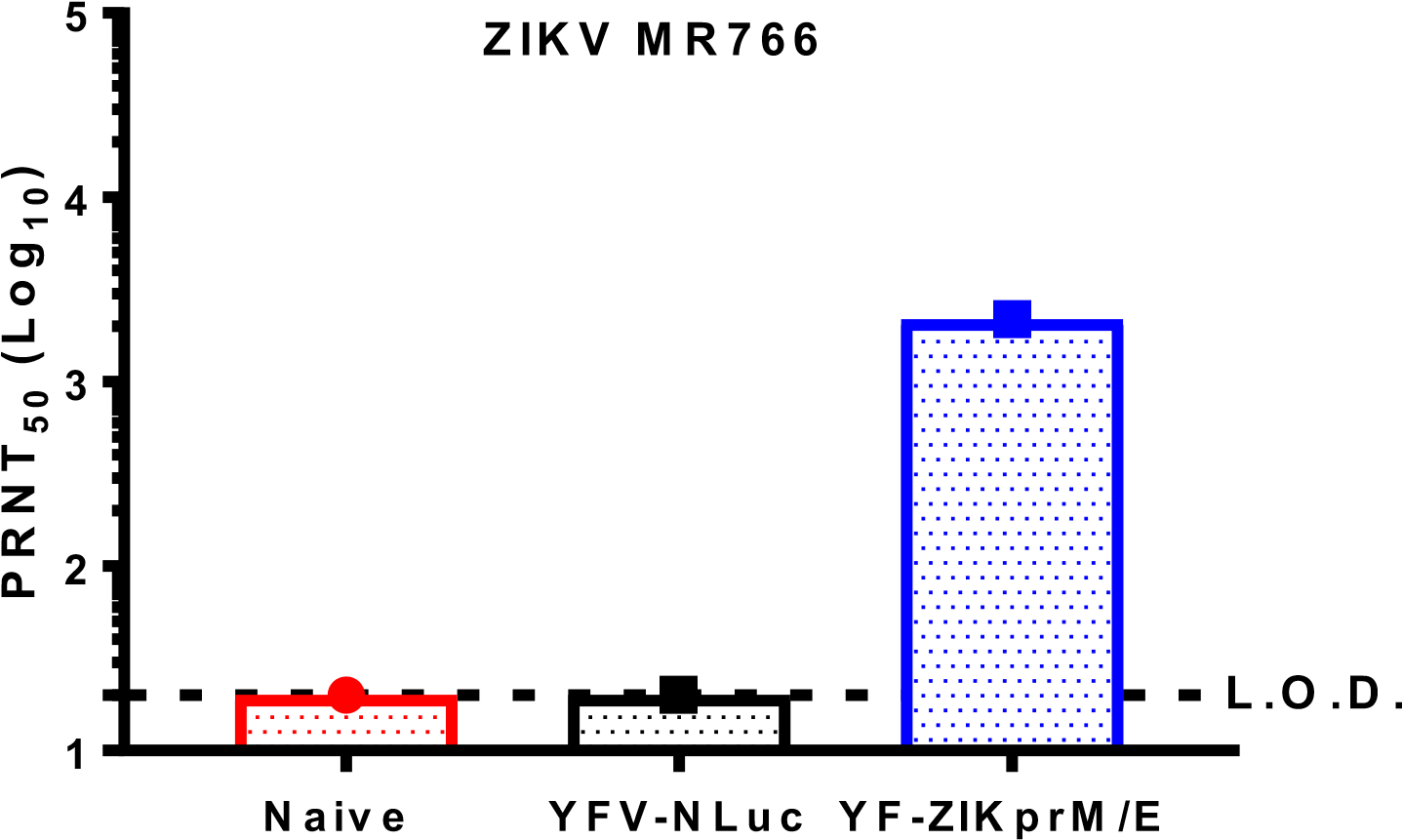


**C**


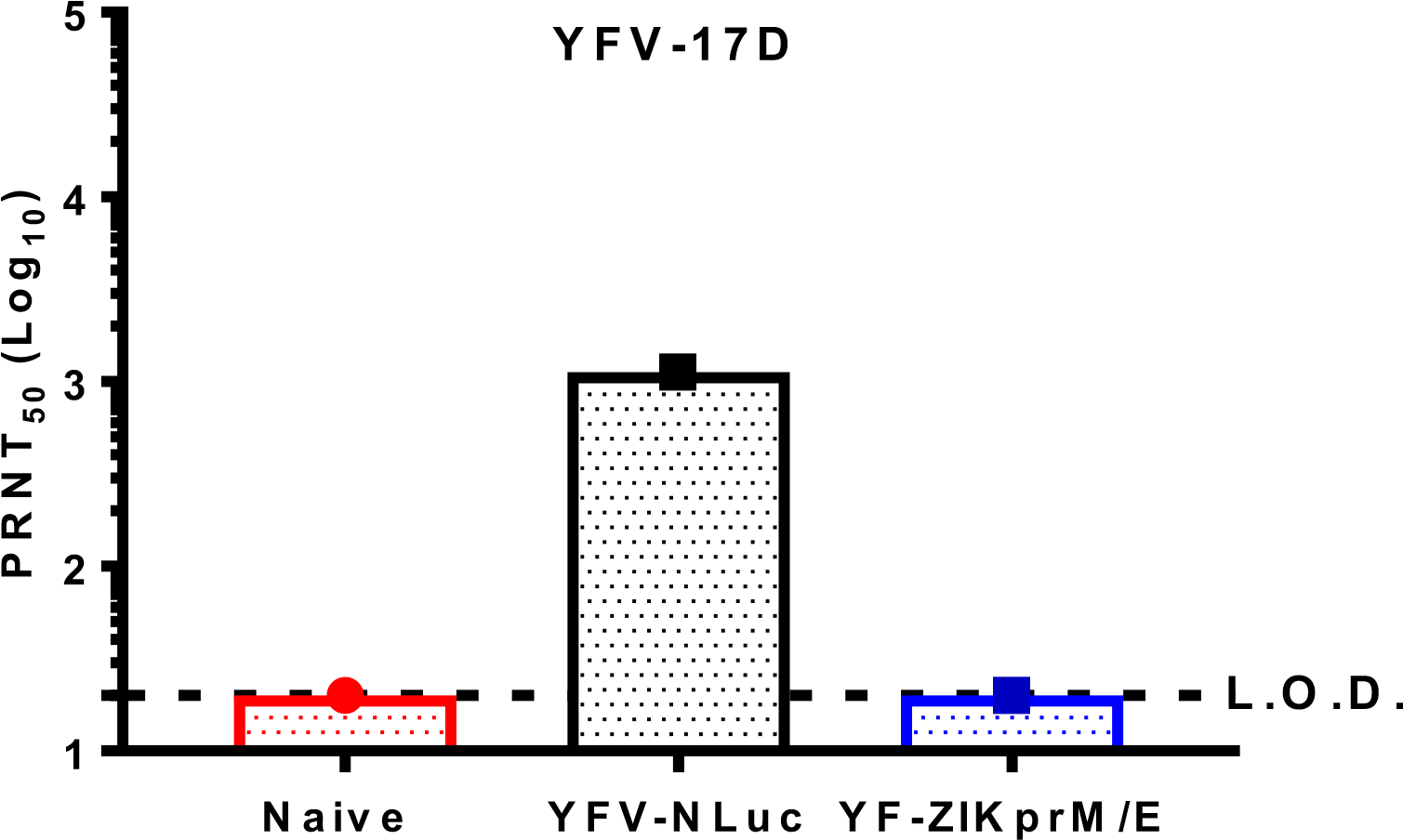


**FIG S3**

**FIG. S3. Characterization of pooled serum prior to transfer to naïve mice and YFV-17D challenge.** Total binding antibody (bAbs) (**A**) and neutralizing antibodies (nAbs) titers assayed for pooled sera (n = 5/group) against ZIKV MR766 (**B**) and YFV-17D (**C**) prior to transfer. Data represent mean values determined in technical replicates (n = 3) each. Dotted line denotes the limit of detection (L.O.D.) of the assay.


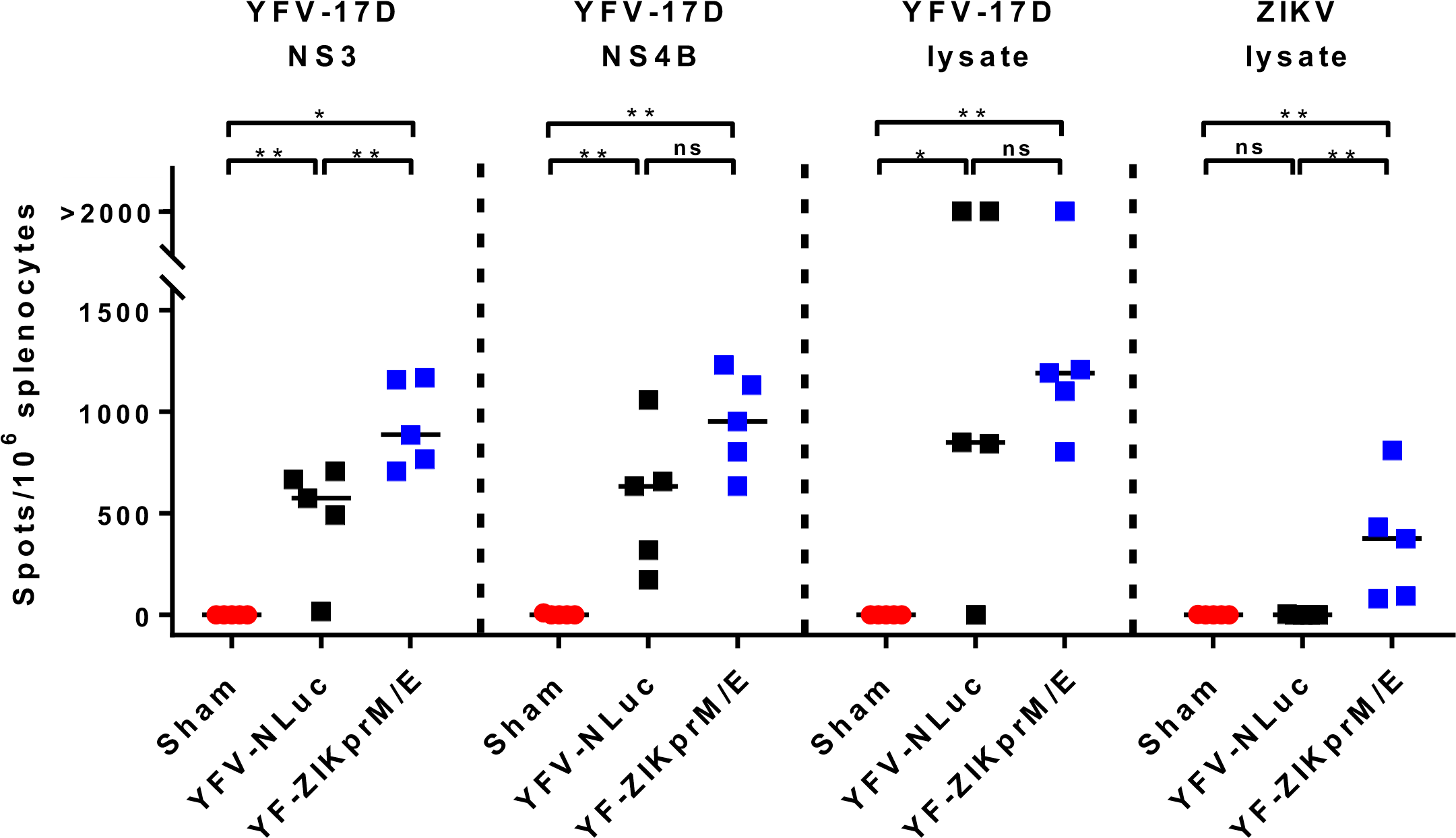


## FIG S4

**FIG. S4. ELISPOT of splenocytes with flavivirus antigens.** *Ifnar*^-/-^ mice were either sham-vaccinated (red circles, n = 5) or vaccinated with YFV-17D-NLuc (black squares, n = 5) or YFV-17D (blue squares, n = 5). Ten weeks postvaccination, mice were euthanized and splenocytes were harvested for *ex vivo* stimulation with cell lysates that had been infected with YFV-17D or ZIKV. Bars represent median values of biologically independent samples (n = 5). Dotted lines represent the background values from sham-vaccinated mice. Differences between groups were quantified by Mann-Whitney two-tailed test. *P*-values <0.05 were considered statistically significant. **P* < 0.05, ***P* < 0.01, ns = nonsignificant.

***ifnar^-/-^* YFV-17D, 5dpi**

**A**


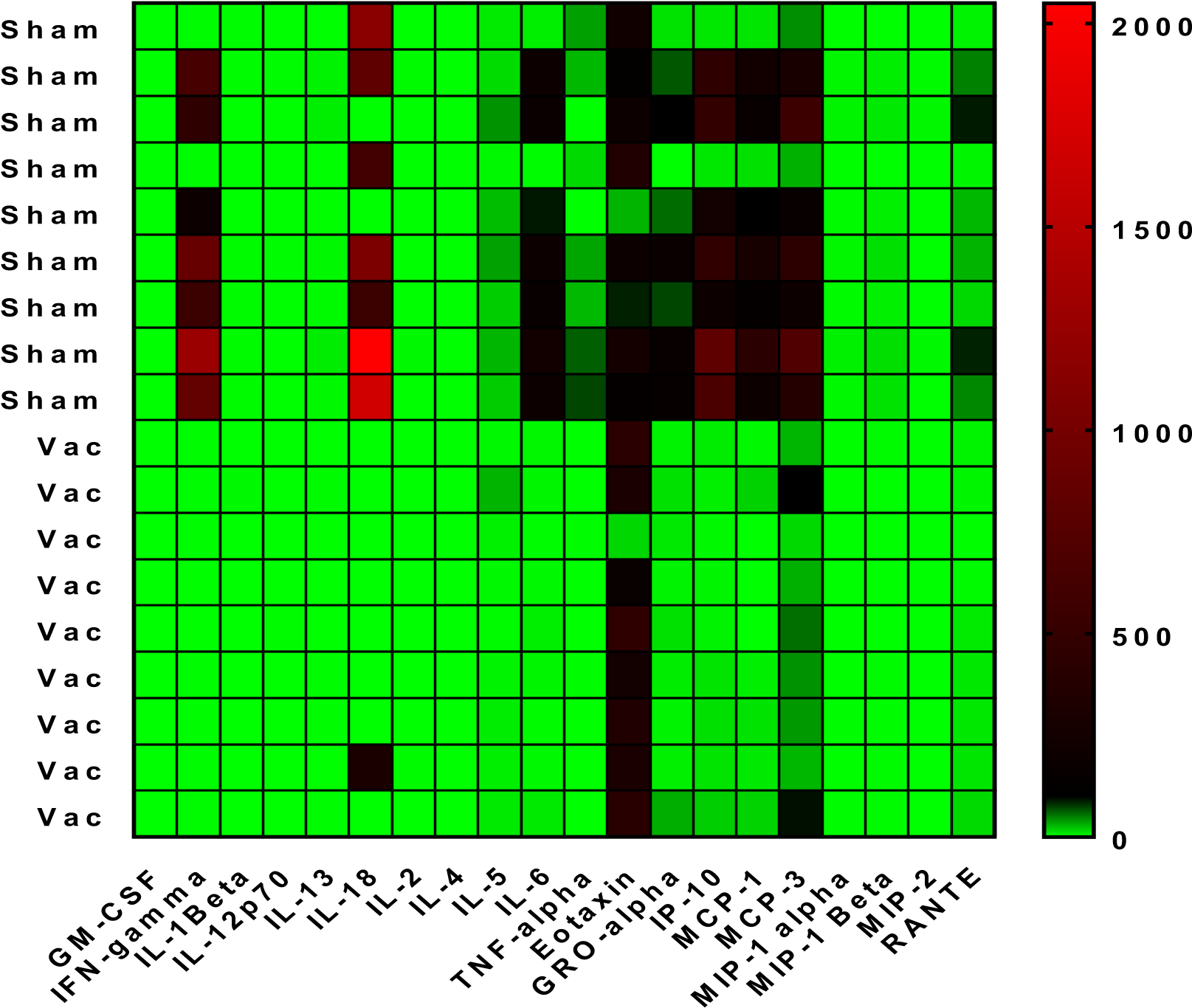


**B C57BL/6 YFV-17D, 5dpi**


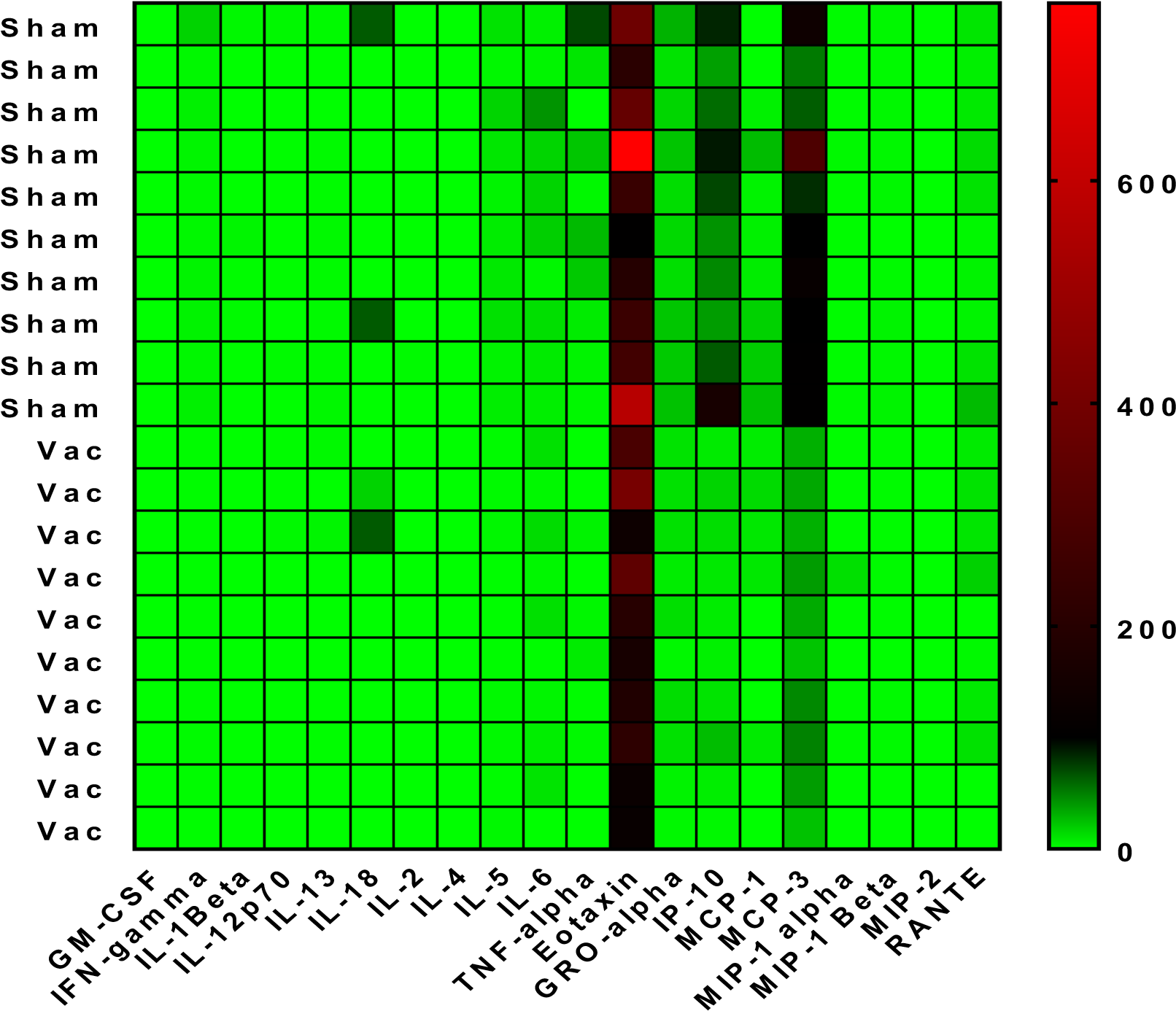


## FIG S5

**FIG. S5. Heat maps of cytokines in different mouse models.** *Ifnar*^-/-^ (**A**) and C57BL/6 (**B**) were either sham-vaccinated (n = 5 – 10) or vaccinated (n = 9 – 10) with YF-ZIKprM/E and challenged i.p. with 1 x 10^4^ PFU of YFV-17D. Mice were bled 5 days post challenge by submandibular puncture and sera were used to quantify cytokines following YFV-17D infection. Data present mean values of individual samples (n = 5 – 10/group).

**Table S1. Primers and probes used for qRT-PCR**

| **Primer/Probe** | **Sequence (5’ - 3’) ^(a)^** | **Source/Targe ^(b)^** | **Size** | **Position** |
| --- | --- | --- | --- | --- |
| YFV Forward | TGGCATATTCCAGTCAACCTTCT | YFV-17D **NS3** | 143 | 4645-4667 |
| YFV Reverse | GAAGCCCAAGATGGAATCAACT |  |  | 4767-4788 |
| YFV-MGB Probe | FAM–TTCCACACAATGTGGCATG–MGB |  |  | 4712-4739 |

^(a)^ Reporter dye (FAM) and TaqMan MGB (minor groove binder) quencher probes ^(b)^ Target sequence in the YFV-17D genomes (GenBank accession no. X03700).

**Table S2. P-values for ELISPOT of splenocytes stimulated with different flavivirus antigens**

|  | **Sham vs YFV-NLuc** | **Sham vs YF-ZIKprM/E** | **YFV-NLuc vs YF-ZIKprM/E** |
| --- | --- | --- | --- |
| **YFV-17D NS3** | **0.0079 | *0.0159 | **0.0079 |
| **YFV-17D NS4B** | **0.0079 | **0.0079 | 0.0952 |
| **YFV-17D lysate** | *0.0476 | **0.0079 | 0.8333 |
| **ZIKV lysate** | >0.9999 | **0.0079 | **0.0079 |
